# In Situ Application of Anti-Fouling Solutions on a Mosaic of the Archaeological Park of Ostia Antica

**DOI:** 10.3390/ma15165671

**Published:** 2022-08-18

**Authors:** Andrea Macchia, Hélène Aureli, Chiara Biribicchi, Antonella Docci, Chiara Alisi, Fernanda Prestileo, Francesco Galiano, Alberto Figoli, Raffaella Mancuso, Bartolo Gabriele, Mauro Francesco La Russa

**Affiliations:** 1 YOCOCU, Youth in Conservation of Cultural Heritage, Via T. Tasso 108, 00185 Rome, Italy; 2 Department of Biology, Ecology and Earth Sciences DIBEST, University of Calabria, Via Pietro Bucci, Arcavacata, 87036 Rende, Italy; 3 Department of Earth Sciences, University of Rome “La Sapienza”, Piazzale Aldo Moro 5, 00185 Rome, Italy; 4 Archaeological Park of Ostia Antica, Via dei Romagnoli 717, 00119 Rome, Italy; 5 Department of Environment, Global Change and Sustainable Development, ENEA, CR-Casaccia, 00123 Rome, Italy; 6 CNR-ISAC, Via Fosso del Cavaliere 100, 00133 Rome, Italy; 7 Institute on Membrane Technology, National Research Council of Italy (CNR-ITM), 87036 Rende, Italy; 8 Laboratory of Industrial and Synthetic Organic Chemistry (LISOC), Department of Chemistry and Chemical Technologies, University of Calabria, Via Pietro Bucci 12/C, 87036 Rende, Italy

**Keywords:** biocides, antimicrobial coating, portable techniques, stone materials, mosaic

## Abstract

Biodegradation is among the most common issues affecting Cultural Heritage stone materials in outdoor environments. In recent years, the application of chemical agents with biocidal activity has been the most usual practice when dealing with biofilm removal. In outdoor environments, the use of these biocides is not effective enough, since the materials are constantly exposed to environmental agents and atmospheric pollutants. Thus, it becomes necessary to protect the surface of Cultural Heritage works with antimicrobial coatings to either prevent or at least limit future colonization. In this study, innovative biocides—both natural and synthetic—were applied on a Roman mosaic located in the Archaeological Park of Ostia Antica to compare their effectiveness in removing the biological degradation affecting it. In addition, an antimicrobial coating called “SI-QUAT” was applied and analyzed in situ. SI-QUAT has recently entered the market for its prevention activity against biocolonization. The biocidal activity of these products was tested and monitored using different analytical portable instruments, such as the multispectral system, the spectrocolorimeter, and the bioluminometer. The analyses showed that promising results can be obtained using the combination of the biocide and the protective effect of Preventol^®^ RI50 and SI-QUAT.

## 1. Introduction

Biodeterioration is among the main issues related to the conservation of Cultural Heritage stone materials in outdoor environments. Biofilms can be found in archaeological sites and hypogean spaces, but also on statues and buildings [1]. Biodeterioration is caused by the formation of active biofilms, due to interacting microbiota growth constituting a complex ecosystem. Contamination depends on different factors, such as environmental conditions—relative humidity (RH%), light, and temperature—and the physicochemical properties of the substrate, i.e., roughness, absorbency, hydrophobicity, porosity, and chemical composition [2]. Biofilms growing on stone monuments generally include photolithoautotrophs, such as algae, cyanobacteria, mosses, and higher plants. As the biomass expands, it releases organic nutrients fostering the growth of other microorganisms, such as chemolithoautotrophic bacteria, chemoorganotrophic bacteria, and fungi [3]. Biogenetic pigments lead to aesthetical damage, causing the formation of stains on the stone artifacts. Furthermore, biofilms produce extracellular polymeric substances (EPS) that induce mechanical degradation due to both the penetration of hyphae and filaments, and the shrinking-swelling cycles of biological colloidal particles in the material’s pores. In addition, it is worth noting that the size and distribution of the pores, moisture circulation, pH, and water permeability of the stone might be altered by the occurrence of biocolonization. It has been demonstrated that the presence of biogenic patinas on exposed surfaces accelerates the accumulation of atmospheric pollutants. Generally, the first step in treating biodeteriorated surfaces is the application of chemical products with biocide properties aiming at directly intervening on the active biofilm. For this kind of application, biocides have to meet specific criteria: they must act against the microorganisms, not be dangerous to the substrate, be long lasting, biodegradable, and non-toxic to both human health and the environment. Nonetheless, conventional commercial products used as biocides with anti-fouling and anti-biological effects, such as Preventol^®^ RI50 (quaternary ammonium salt-based biocide), are often toxic. Additionally, their dispersion in the surrounding environment cannot be controlled [2,4,5,6]. For this reason, in the last decade, the search for non-toxic and eco-friendly reagents to remove and control biodeteriogens on Cultural Heritage artifacts is ever-growing [7,8,9,10]. Indeed, natural-based agents have been tested as alternative biocides, such as plant extracts and essential oil mixtures [11,12,13]. After carrying out biocidal treatments, it is necessary to control the environmental and microclimatic conditions that might promote the microflora growth to limit and prevent future biodeterioration processes. Although it is easy to control indoor environments, this process is not possible when dealing with outdoor-exposed Cultural Heritage, such as archaeological sites. Indeed, it is well known that the biocidal effect does not last long if the surface of the material is not protected, for example using roofs or seasonal coverings to limit the exposure to rainwater. Furthermore, recurrent biocide treatments can induce the resistance of the microorganisms [14]. In situ conservation requires both active and preventive conservation. For this reason, after the cleaning process, the application of an appropriate microbial-resistant protective product on the stone surface exposed in an open environment is suggested [15]. Protective coatings may work repelling the microorganisms, namely avoiding their colonization on the surface, or killing them. This second action is generally achieved by adding biocides—such as silver, quaternary ammonium compounds, and active chlorine—to the protective film [16]. Indeed, for a long time, water repellants have been used as retardants despite their properties might interfere with the biocide action, but also develop disfiguring patterns and have short effects [17]. The application of a biocide on a surface treated with a water-repellant product can interfere with hydrophobicity. This hydrophilization depends on the type of product, the concentration, and the amount applied on the material. Consequently, the use of a water repellant on a stone substrate can lead to ineffective results as it may block the interaction between water droplets and any trace of the treated biocides [18]. In addition, water repellants were reported to be just effective against algae and lichens but not against fungi, especially black fungi [19]. Hence, antimicrobial polymeric films based on quaternary ammonium salts have recently gained interest in the development of new biocide protective systems. They combine the ability of quaternary ammonium salts to exchange ions between the membrane cell and positively charged groups, causing the disintegration of the membrane and, consequently, the cell death with polymerizable function [20]. Polymers are non-volatile and low toxic. They also have minimal permeability, potentially long-term effects, and increased stability [21]. Their combination with antimicrobial agents, such as quaternary ammonium silane (QAs) forming QA copolymer coatings, has been demonstrated to be less toxic and have better durability.

**Figure 1:**
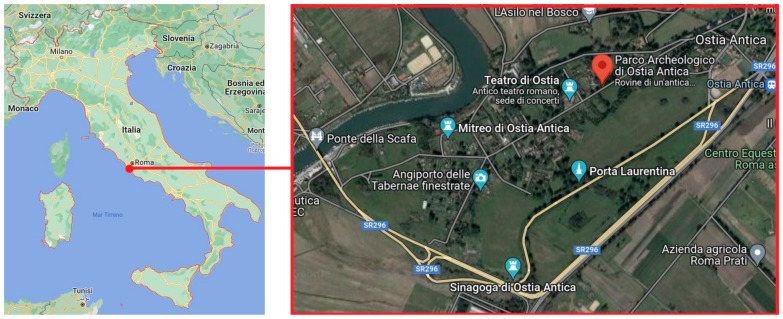
Map of Italy indicating the location of Ostia Antica.

Thus, following our previous research on essential oils as biocides, the research aimed at evaluating the biocide action of different products applied in situ, specifically on the surface of a biodeteriorated Roman mosaic located in the XIX room of the “Insula delle Muse” in the Archaeological Park of Ostia Antica (Figure 1) [22]. Their effectiveness was studied using different analytical techniques, such as ultraviolet-induced luminescence (UV), spectrocolorimetry, and bioluminometry. Furthermore, SI-QUAT, a quaternary ammonium salt-based hybrid organic-inorganic agent consisting of dimethyl octadecyl (3-(trimethoxysi[y]propyl) ammonium chloride (DTPCA), was applied on an area previously treated with Preventol^®^ RI50 and mechanically cleaned. Even though it is not a biocide, SI-QUAT was tested as a new antimicrobial active surface coating to evaluate its ability to limit new colonization by comparing the protected area to another one treated with Preventol^®^ RI50 alone and mechanically cleaned. It is biologically active on inorganic surfaces since it links to the microbes’ long molecular chains by ionic association and lipophilic attraction, breaking their cell walls and, therefore, killing them [23]. The lysis of the membrane cells of model Gram-positive and Gram-negative bacteria by the application of this product was reported by Speier and Malek [24]. SI-QUAT controls the micro-biological contamination without releasing toxic compounds, as it has a non-leaching behavior and forms a water repellent barrier [25]. Data about the effects of this new product, when combined with a biocide treatment, are still missing and no case studies have been reported. For this reason, the research aimed at providing further information about the application of SI-QUAT protective coating on outdoor-displayed Cultural Heritage.

## 2. Materials and Methods

Six different biocide products—both synthetic and natural based—were tested on a white and black mosaic located in the XIX room of “Insula delle Muse” (red area), in the Archaeological Park of Ostia Antica in November 2020. As reported in Table 1, Preventol^®^ RI50, AUTEAB, Biotersus, Bionature, Essenzio, and Liq were used. Apart from AUTEAB, which was applied on a membrane, the other biocides were applied by brushing.

Preventol^®^ RI50 (CTS), a traditional quaternary ammonium salt-based biocide diluted with distilled water at 3% *v*/*v*, was applied as a reference (zones 1–2, Table 1). A novel green product called BioTersus (Exentiae)—a mixture of different essential oils (*Cinnamonum zeylanicum* (0.25% *v*/*v*), *Eugenia caryophyllata* (0.5% *v*/*v*), *Corydothymus capitatus* (0.4% *v*/*v*), and the surfactant Tween^®^20 (0.3% *v*/*v*)—was tested diluted with distilled water at a concentration of 1.3% *v*/*v*, as suggested by technical data sheets (zone 8, Table 1) [26]. A new natural extract called BioNature, which is a derivative of *Rosmarinus officinalis*, *Allium sativum*, and *Capsicum annum* proposed by YOCOCU APS, was applied in a hydro-alcoholic solution (zone 5, Table 1). BioNature is based on natural extracts with promising biocide effects, as reported in the literature [6,27,28,29,30]. Essenzio (IBIX) is an extract derived from *Thymus vulgaris* and *Origanum vulgare*. It was applied as it is on the mosaic’s surface using a soft brush (zone 3, Table 1) [31]. An alcoholic extract derived from licorice, now on called LIQ, was supplied by Trifolio-M. It was used at the concentration of 5% (*v*/*v*) in water (zone 7, Table 1) [32]. AUTEAB, an acryloyloxyundecyltriethylammonium bromide-based chemical, was supplied by YOCOCU-UNICAL-CNR [33,34]. It was applied using a membrane as a supporting agent following two different procedures, namely as after the synthesis and after three washing cycles in distilled water. The washing with distilled water aimed at removing the AUTELAB excess. An anti-fouling, anti-bacterial, and antiviral active coating, formulated by YOCOCU in collaboration with the University of Calabria, called SI-QUAT (Affix-Labs), a DTPAC agent, was applied using a soft brush on a zone cleaned and treated with Preventol^®^ RI50 a week earlier. We would like to point out that SI-QUAT was not used as a biocide, but instead as a protective agent to prevent the formation of new biofilms. For this reason, its performance was evaluated after two weeks and after 7 months from the treatment.

**Figure 2:**
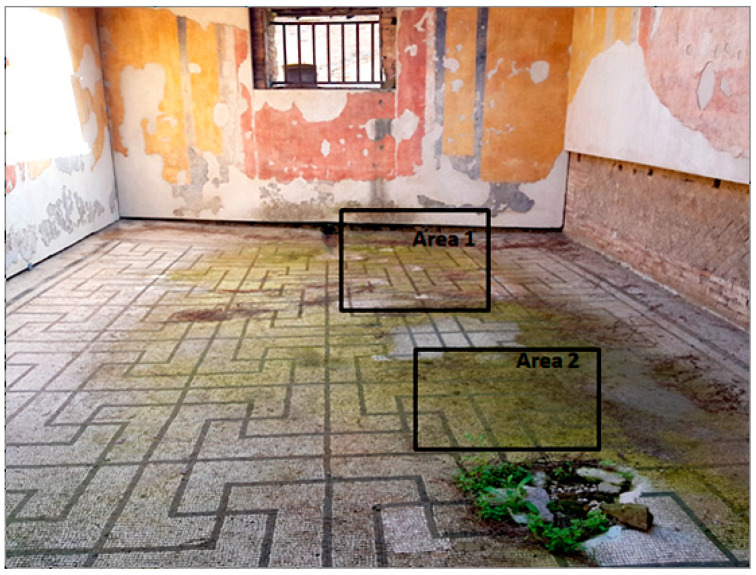
XIX room of “Insula delle Muse” at the Archaeological Park of Ostia Antica. The figure shows the treated Areas 1 and 2 of the biodeteriorated mosaic.

The selected biocides were applied on the green patina present on the mosaic. It was mainly characterized by cyanobacteria, Chlorophyta (photosynthetic prokaryotes), and green algae (Chlorella) [22,35]. Since the mosaic showed different levels of biodeterioration, the selected products were tested on two significant regions of the mosaic’s surface, from now on denominated Area 1 and Area 2 (Figure 2). Indeed, as it can be seen both macroscopically and using an optical microscope, Area 1 was characterized by minor colonization when compared to Area 2, because the latter is constantly exposed to the water flow coming from the upper roof [22]. Both Area 1 and Area 2 were further divided into nine areas, denominated zones 1, 2, 3, 4, 5, 6_1, 6_2, 7, and 8 (Table 1), from now on, respectively, called z1, z2, z3, z4, z5, z6_1, z6_2, z7, and z8. The reagents were applied on each zone, then covered with a transparent polyethylene film and left there for a week.

One of the nine zones was cleaned both mechanically and with Preventol^®^ RI50, which was used as a reference. The same procedure was followed in the zone on which the SI-QUAT was applied as a protective agent.

After a week, the biocides were applied a second time following the same steps to achieve stronger biocide action. After the second treatment, the biological patina was removed. The surfaces of areas Area 1 and Area 2 were cleaned mechanically and by brushing using distilled water, brushes, and eventually sponges to remove possible residues. The surfaces of Area 1 and Area 2 were covered using water-resistant DELTA LITE PLUS sheets (BSN Medical, Delta-Lite) to protect the treated areas from environmental and atmospheric agents [36].

In this study, multiple analytical methods were used to test the effectiveness of the selected biocides on Area 1 and Area 2. Specifically, ultraviolet-induced luminescence (UVL) was exploited to evaluate the state of the mosaic’s surface, the extension of the biological patina, and the efficacy of the biocides by comparing the images taken before and after the treatment. UVL was carried out using two Madatec spotlights at wavelength of 365 nm (UV) to detect the visible light re-emitted by the chlorophyll of the biodeteriogens. Pictures were taken using a Madatec multispectral system supported with a Samsung NX50028.2 MPBSICMOS camera. Two filters were used to observe the fluorescence, namely the HOYA UV-IR filter cut 52 and the Yellow 495 52 mm F-PROMRC022 [37,38]. Spectro-colorimetric analysis was performed before and after the treatment using a portable spectrophotometer Y3060 3 nh, with a D65 illuminant, 8 mm size aperture, in the SCI mode (Specular Component Included). The variation of the parameters L*, a*and b* was calculated evaluating the distance between the values acquired on the areas before and after the application of the biocides. The spectra (400–700 nm) were measured. Data were then analyzed through the CIELab color system. An increase in Δb* and a decrease in ΔL* and Δa* values can be related to recolonization processes. This phenomenon can be observed when the treated surface becomes darker and more green-yellowish due to the growth of phototrophic microorganisms [36,39]. Positive values of ΔL* and Δa* with a decrease in Δb*are related to decolonization processes, thus indicating the biocide action of the treatment. Adenosine triphosphate (ATP) analysis was carried out to examine the emitted fluorescence of the chlorophyll, the chromatic parameters of the mosaic’s surface, and the presence of ATP. The count of ATP was used to gain information on the metabolic activity of microorganisms or organic residues present on the two areas after the cleaning treatments. Metabolic activity was assessed rubbing a sterile cotton swab present on the surface of the two areas to collect particles after the treatments. A portable bioluminometer LumitesterPD-30 (Kikkoman) and LuciPacPen AQUA was used. ATP analysis was performed on a non-treated area to be used as a reference of the biological contamination of the mosaic.

Each investigation was performed three times both before and after the whole treatment to reduce the uncertainty of the analyses. Data are reported as the means ± standard deviation (SD). Statistical analyses were performed by Student’s *t* Test or one-way ANOVA, followed by Tukey’s Multiple Comparison Test (Homogeneous Variances). Statistical significance was set at *p*-value < 0.05.

Data on climate conditions during the period of the experimentation are presented in Table 2 to provide comprehensive information about the external factors involved in the research.

Temperature and rainfall data on the days when the treatments and analyses were carried out are also reported (Table 3).

## 3. Results

### 3.1. Multispectral Analysis

Photographs were taken in visible reflectance imaging before the treatment, after two weeks from the application, after the cleaning tests, and seven months later (Table 4). Area 1 showed a dark greenish-brownish dry patina if compared to the dark-green biofilm of Area 2. In the rest of this paper, the application of SI-QUAT on the area treated using Preventol^®^ RI50 and mechanical action will be addresses as “SI-QUAT” or zone “z4”. Based on macroscopic observation, after the application of the products, the best results were obtained on Area 1 AUTEAB (z6_1), followed by Esssenzio (z3), washed AUTEAB (z6_2), Preventol^®^ RI50 (z2), LIQ (z7), Biotersus (z8), and Bionature (z5). Area 2 showed a stronger biocide action compared to Area 1. The best results were obtained on z6_1, z8, which were, respectively, treated with AUTEAB, and Biotersus, followed by z5 (Bionature), z6_2 (washed AUTEAB), z7 (LIQ), and z3 (Essenzio). After the cleaning treatment, the green biological patinas were completely removed, revealing the original white color of the mosaic tesserae, and multispectral imaging was performed once again. Nevertheless, some residues characterized by spread reddish stains were detected on Area 1. They appeared to be especially present on the central region of Area 1, namely z5, z8, and z2. The strongest cleaning action in Area 1 can be described as z8 > z3 > z6_1 > 6_2 > 5 > 7 and 6_2. As to Area 2, the same effect can be observed in z2, z8, z6_1, z5, z3, z7, and z6_2. After one month from the cleaning process, it was possible to observe biological recolonization on every treated zone. However, z2 which was treated with Preventol^®^ RI50, showed slighter colonization compared to the others. Additionally, it is possible to observe that the area treated with SI-QUAT showed a slightly lesser degree of colonization after 7 months if compared with z1.

All the zones of Areas 1 and 2 showed strong emitted light under UVL before the application of the biocides, due to the presence of chlorophyll molecules contained in the biodeteriogens (Table 5). The UV fluorescence appeared to be particularly intense in z5, z2, z3 of Area 1 and in z6_1, z7, z8, z6_2, z5, z2, and z3 of Area 2, confirming the hypothesis about the parts that seemed to have the strongest biocontamination. After the cleaning treatment, the surface of both the areas did not show emitted light, since the microorganisms were probably removed by mechanical cleaning. The best cleaning efficacy on Area 1 was reached using Bionature (z5), and AUTEAB (z6_1), followed by partial efficacy on z2, z6_2, z7, z8, and z3 Table 6). As to Area 2, the best results were obtained in z6_2, and z8. Partial efficacy was observed on z2, z7, z3, z5, and z6_1 (Table 6). It is to notice that after the second application—before the cleaning process—the strongest reduction in remitted light for Area 1 was detected in z2, z5, and z6_1 followed by z3, z8, z6_2, and z7. A similar trend was reported in Area 2 were z2, and z6_1 showed the lowest emission, followed by z3, z5, z8, z6_2, and z7. Eventually, two weeks after the treatment, the area treated with SI-QUAT (z4) emitted almost the same degree of UV fluorescence with respect to zone z1.

### 3.2. Spectrocolorimetric Analysis

After the application of the biocides (2 weeks), the greatest colorimetric variation compared to the untreated zone and the least colorimetric variation compared to the cleaned zone was achieved by AUTEAB (z6_1), followed by Biotersus (z8), Bionature (z5), Essenzio (z3), Preventol^®^ RI50 (z2), LIQ (z7), and washed AUTEAB (z6_2) for Area 1 (Table 7). As to Area 2, AUTEAB (z6_1) is followed by Preventol^®^ RI50 (z2), Biotersus (z8) and Essenzio (z3), Bionature (z5), washed AUTEAB (z6_2), and LIQ (z7).

Z6_2 of Area 1 (washed AUTEAB) and z6_1 (AUTEAB) of Area 2 showed consistent variation. Minor differences were observed on Area 1, respectively, by using Preventol^®^ RI50 (z2), LIQ (z7), Essenzio (z3), and Bionature (z5), AUTEAB (z6_1), and Biotersus (z8). As for Area 2, partial efficacy was recorded on z6_2, z2, z8, z5, z3, and z7, where washed AUTEAB, Preventol^®^ RI50, Biotersus, BioNature, Essenzio and LIQ were repectively applied.

Colorimetric analysis was performed on z4 of both Area 1 and Area 2 to evaluate the efficacy of SI-QUAT in maintaining the color of the cleaned surface. It presented the lowest variation compared to the cleaned zone (z1).

**Figure 3:**
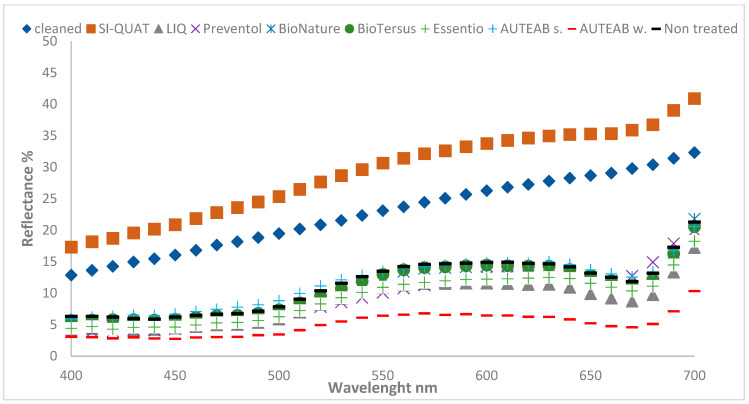
Full spectrum color measurement of the treated zones in Area 1. Standard deviation 0.5 < SD < 3.2.

Figure 3 shows the colorimetric spectrum acquired on each treated zone of Area 1 compared to the spectrum of a cleaned zone (blue marks). The washed AUTEAB confirmed to be the best product among biocides. The other treatment showed analogous trend. Nevertheless, the chlorophyll absorption peak can be seen in every spectrum collected from the treated zones. In addition, SI-QUAT (z4) presented the most similar trend to the cleaned zone (z1), attesting its preservation effectiveness.

**Figure 4:**
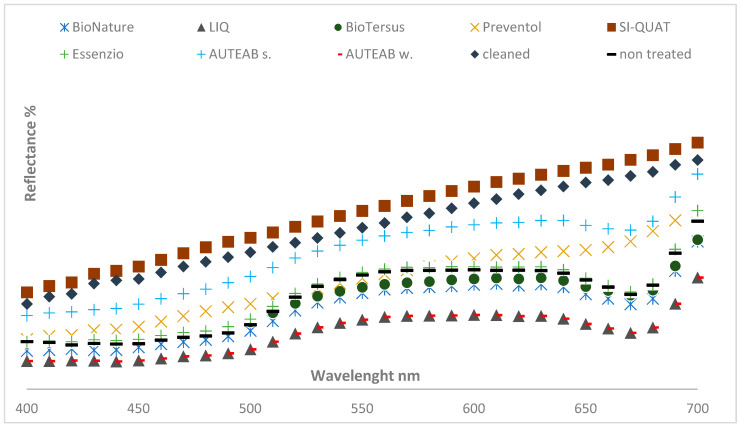
Full spectrum color measurement of treated zones in Area 2. Standard deviaton 0.5 < SD < 3.2.

The best action in Area 2 was recorded by Preventol^®^ RI50 (z2), and AUTEAB (z6_1) (Figure 4). It can be also observed that SI-QUAT showed better results if compared to z1, attenting its preservation ability.

### 3.3. Adenosine Triphosphate (ATP) Test

**Figure 5:**
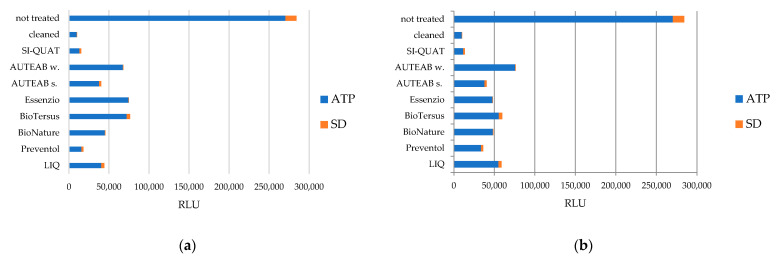
Histograms reporting the measures of ATP (in blue) collected after the treatment of each tested zone of Area 1 (**a**) and Area 2 (**b**) and a non-treated zone (**a**,**b**).

In both Area 1 and Area 2, the strongest biocide action was obtained using Preventol^®^ RI50 (z2) and AUTEAB (z6_1), showing the lowest residual metabolic activity (Figure 5a,b). A discrete amount of ATP was detected on different zones, in the following descending order: LIQ > BioNature > washed AUTEAB > Biotersus > Essenzio of Area 1 (Figure 5a). As to Area 2, the values of ATP are slightly higher, indicating minor biocidal effect (Figure 5b). This phenomenon was especially observed in the following zones in ascending order (from minor to major biocide activity): z8, z3, z6_2, z5, and z7 where Biotersus, Essenzio, AUTEAB washed, BioNature, and LIQ were, respectively, applied. The area treated with SI-QUAT showed a similar trend if compared with z1.

### 3.4. Analysis for the Evaluation of Products’ Long-Lasting Trend

**Figure 6:**
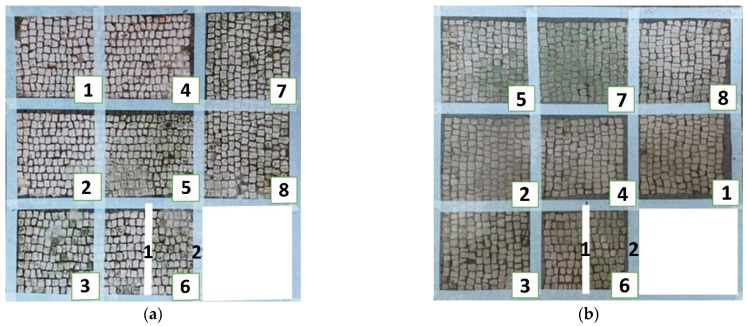
Images of Area 1 (**a**) and Area 2 (**b**) after seven months from the cleaning treatment.

The long-lasting trend of the biocides was examined after seven months (Figure 6). At macroscopic observation, slightly visible green biological growth was observed on the areas. On Area 1, z4, z6_1, and z2, where Preventol^®^ RI50, SI-QUAT, and AUTEAB were, respectively, applied, appeared to be less contaminated. On z4, z8, and z2 of Area 2, where SI-QUAT, Biotersus and Preventol^®^ RI50 were tested, no visible re-growth was observed. The greatest performance was obtained on z4 of both Area 1 and Area 2, where the product SI-QUAT was applied, confirming its preservation ability.

**Figure 7:**
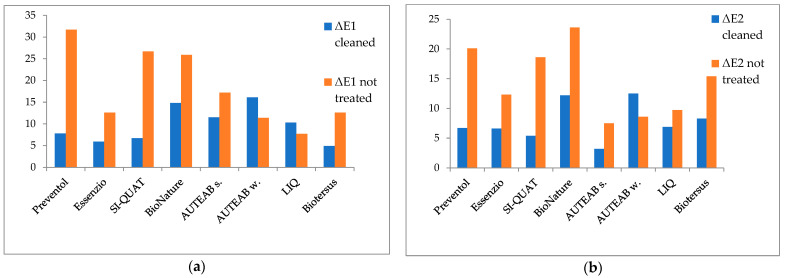
Histogram reporting the ΔE* parameter associated to the cleaned zone and the non-treated zone of Area 1 (**a**) and Area 2 (**b**). Measures taken seven months after the cleaning treatment. Standard deviation 0.5 < SD < 3.2.

The chromatic parameter ΔE* of Area 1 and Area 2 after seven months from the cleaning procedure are reported in Figure 7. Z8 of Area 1 (Biotersus) showed the best long-lasting effect. Partial effectiveness was reported in the areas treated using Essenzio (z3), SI-QUAT (z4), and Preventol^®^ RI50 (z2), followed by LIQ (z7), AUTEAB (z6_1), Bionature (z5), and washed AUTEAB (z6_2). As to the ΔE* measured on the non-treated zone, the best action was reached by the product Preventol^®^ RI50 (z2), SI-QUAT (z4), and Bionature (z5). Slighter efficacy was observed in descending order on z6_1, z8, z3, z6_2, and z7, namely the areas treated with supported AUTEAB, Biotersus, Essenzio, washed AUTEAB, and LIQ. Z6_1 of Area 2 (application of AUTEAB) presented the smallest variation compared to the reference cleaned zone, followed by SI-QUAT (z4), Preventol^®^ RI50 (z2), Essenzio (z3), and LIQ (z7). Higher variations were observed in z8, z5 and z6_2. When compared to the non-treated zone, the best result was reached by Bionature (z5), followed by Preventol^®^ RI50 (z2), and SI-QUAT (z4). Minor variation was detected in descending order in the areas where Biotersus (z8), Essenzio (z3), LIQ (z7), washed AUTEAB (z6_2), and AUTEAB (z6_1) were applied. Overall, the best treatments resulted to be Preventol^®^ RI50 (z2) and SI-QUAT (z4) on all the examined areas, as they showed the smallest ΔE* related to the cleaned zone and the higher difference when compared to the non-treated zone. Biotersus, Essenzio, Bionature, and supported AUTEAB showed partial efficacy in Area 1, followed by z7 and z6_2, which were treated LIQ and AUTEAB. As to Area 2, the effectiveness decreased in descending order on z6_1, z8, z3, z5, z7, and z6_2, where supported AUTEAB, Biotersus, Essenzio, BioNature, LIQ, and washed AUTEAB were applied.

## 4. Discussion

Table 8 summarized the best results of each implemented analytical technique on each zone of Area 1 and Area 2 to define the strongest biocide action among the selected products. Preventol^®^ RI50 (z2) was reported to be the most efficient biocide on Area 1, meanwhile, the best biocide action was obtained using AUTEAB (z6_1) on Area 2. Additionally, the greatest performance was obtained in z4 of both the areas, namely where the product SI-QUAT was applied.

It has to be noted that, even though the effectiveness of natural biocides appears to be lower when compared to traditional biocides, some of them showed promising results. Thus, they should be considered as a potentially suitable alternative to more toxic products. Additionally, the synergy of a biocide and antimicrobial protective coating provided encouraging perspectives. The combination of a biocide and a protective film could lead to a reduced need for continuous cleaning treatments for the removal of biological patinas using conventional toxic biocides. This topic should be further examined by carrying out accurate long-lasting evaluations, taking into account all the factors which may influence the performance of those treatments on outdoor-displayed Cultural Heritage, namely weather and environmental conditions. Furthermore, an in-depth investigation should be focused on finding a standard method to evaluate the effectiveness of the biocides and the protective agent (SI-QUAT) on biodegradated surfaces of different materials. Eventually, the protective effectiveness of SI-QUAT should be studied further with other natural biocides.

## 5. Conclusions

The research focused on comparing the biocide action of several products, both natural and synthetic, based on different reacting agents. Biotersus, LIQ, Essenzio, and Bionature, which are based on essential oils and extract of plants, were tested. They were also compared to AUTEAB and Preventol^®^ RI50 (quaternary ammonium salt based), the most common biocides in the market and the most widely used in the Cultural Heritage field. Furthermore, the protective activity of an antimicrobial coating, called SI-QUAT, was tested on a zone previously treated using Preventol^®^ RI50 to evaluate its effectiveness in preventing re-colonization after the biocide application. This study aimed at gaining further information about this protective film, because it has never been tested in situ on Cultural Heritage. Therefore, among the goals of the research was to bridge the lack of data in the literature. Tests were carried out on the surface of a roman mosaic located in the XIX room of “Insula delle Muse” at the Archaeological Park of Ostia Antica and covered by a biofilm. After the products were applied twice on the areas of investigation, the biofilm was completely removed. As expected, each treatment expressed variable biocide action on the microorganisms forming the biofilm. The investigation revealed the different levels of efficacy of the chosen products. Weak results were mostly reported by the application of the natural biocides, among which Biotersus obtained the best efficacy, whereas the treatments made using the chemical biocides, which are considered to be toxic, showed moderate and temporary action on the biological patina and, in this case, the best action was reached by membrane-supported AUTEAB. The results obtained on zone 4 of both Area 1 and Area 2, where the surface was cleaned using the biocide Preventol^®^ RI50 and then protected with SI-QUAT, were particularly remarkable, attesting the need for a protective coating able to act as a preventive agent.

## Figures and Tables

**Table 1:** Products used in the experimentation and their zone of application.

Zone	Products	Composition	pH
1 (mechanically cleaned)	Preventol^®^ RI50	Quaternary ammonium salts	8.02 ± 0.3
2	Preventol^®^ RI50	Quaternary ammonium salts	8.02 ± 0.3
3	Essenzio	Extract of *Thymus Vulgaris* and *Origanum Vulgare*	8.7 ± 0.5
4	SI-QUAT	DTPAC	7.5 ± 0.4
5	BioNature	Extract of *Rosmarinus Officinalis, Allium Sativum* and *Capsicum Annum*	8.3 ± 0.5
6	AUTEAB (1. Supported; 2. Washed)	Acryloyloxyundecyltriethylammonium bromide	n.d.
7	LIQ	*Glycyrrhiza Glabra*	5.4 ± 0.5
8	Biotersus	*Cinnamonum Zeylanicum*, *Eugenia Caryophyllata*, *Corydothymus Capitatus*, Tween^®^20	5.02 ± 0.3

**Table 2:** Climate conditions during the period of the experimentation. AT = Average Temperature; Tm = minimum Temperature; TM = maximum Temperature; R = maximum Rainfall; RH = relative humidity; WS = Wind Speed; DR = Days of Rain; DS = Days of Snow; DSt = Days of Storm; DF = Days of Fog.

Date	AT	Tm	TM	R	RH	WS	DR	DS	DSt	DF
11/2020	13.3 °C	9 °C	18.1 °C	38.3 mm	77.50%	8 km/h	6	0	0	0
12/2020	9.4 °C	5.7 °C	13.6 °C	70.7 mm	80.30%	10.7 km/h	18	0	2	2
01/2021	7.7 °C	3.6 °C	11.3 °C	78 mm	75.80%	12.9 km/h	15	0	6	0
02/2021	10.4 °C	5.8 °C	15.4 °C	28 mm	73.20%	10 km/h	7	0	0	4
03/2021	10.5 °C	5.4 °C	15.5 °C	61 mm	65.90%	10.2 km/h	9	0	1	0
04/2021	12.8 °C	7.8 °C	17.4 °C	61.9 mm	68.50%	10.9 km/h	19	0	3	3
05/2021	17.5 °C	12.8 °C	22.1 °C	11.7 mm	66.70%	11.8 km/h	11	0	0	1
06/2021	23.8 °C	18.2 °C	28.9 °C	39.3 mm	61.40%	9.3 km/h	7	0	7	0

**Table 3:** Temperature and rainfall data on the specific days of the treatments and analyses. AT = Average Temperature; Tm = minimum Temperature; TM = maximum Temperature; R = maximum Rainfall.

Date	AT	Tm	TM	R
Application of Biocides (5 November 2020)	18.3 °C	16 °C	21 °C	0 mm
Two weeks after the treatment (19 November 2020)	14 °C	9 °C	20 °C	0 mm
Cleaning treatment (19 November 2020)	14 °C	9 °C	20 °C	0 mm
Seven months after the treatment (14 June 2021)	26 °C	21.2 °C	30 °C	0 mm

**Table 4:** VIS Images of Areas 1 and 2 before the biocide treatments, two weeks after the application and 1 month after the cleaning procedure. Legend of the figures in the table: (1) Preventol^®^ RI50 (cleaned area), (2) Preventol^®^ RI50, (3) Essenzio, (4) SI-QUAT, (5) Bionature, (6_1) AUTEAB supported, (6_2) AUTEAB washed, (7) LIQ, and (8) Biotersus.

Period	Area 1	Area 2
Before the treatment	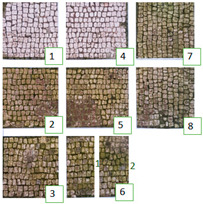	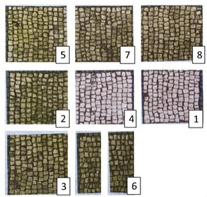
Two weeks after the treatment	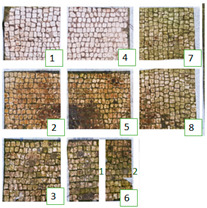	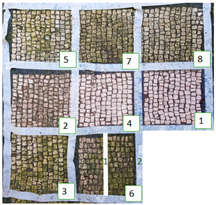
After the cleaning treatment	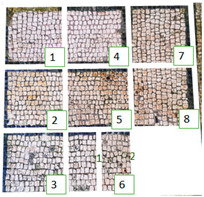	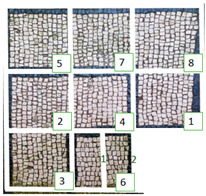
After 7 months	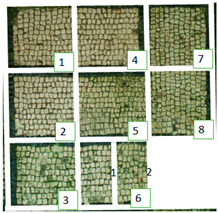	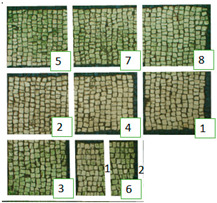

**Table 5:** UV images of Areas 1 and 2 before the biocide treatment, after two weeks from the applications and after the cleaning procedure in UVL. Legend of the figures in the table: (1) Preventol^®^ RI50 (cleaned area), (2) Preventol^®^ RI50, (3) Essenzio, (4) SI-QUAT, (5) Bionature, (6_1) AUTEAB supported, (6_2) AUTEAB washed, (7) LIQ, and (8) Biotersus.

Period	Area 1	Area 2
Before the treatment	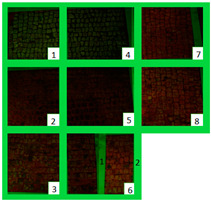	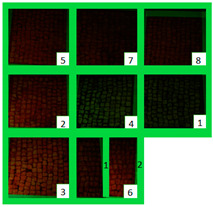
Two weeks after the treatment	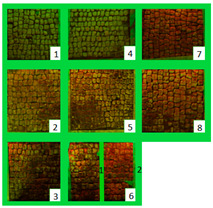	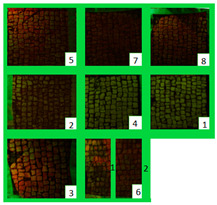
After the cleaning treatment	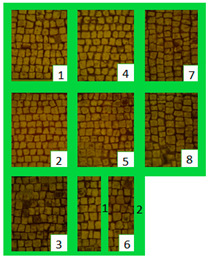	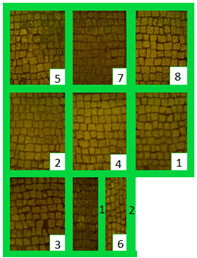

**Table 6:** The level of efficacy of each biocide treatment for Area 1 and Area 2. * indicates low effectiveness, ** medium efficacy, and *** strong action.

Efficacy	Preventol^®^ RI50	Essenzio	BioNature	AUTEAB s.	AUTEAB w.	LIQ	Biotersus
AREA 1	**	*	**	**	*	*	*
AREA 2	**	**	*	***	**	*	**

**Table 7:** Colorimetric parameter ΔE* of each zone of Area 1 and Area 2 calculated as referred to the treated surface and to the non-treated. Standard deviation 0.5 < SD < 3.2.

**Area**	**Parameter**	**Period**	**z2**	**z3**	**z4**	**z5**	**z6.1**	**z6.2**	**z7**	**z8**
1	ΔE1 (cleaned)	After application	25.2	24.8	3.43	24.2	20.3	32.9	25.4	21.8
	ΔE1 (non-treated)	After application	4.5	3.5	20.7	3.5	2.3	13.7	4.0	1.3
2	ΔE2 (cleaned)	After application	11.2	13.8	2.2	15.3	5.4	20.5	20.5	13.8
	ΔE2 (non-treated)	After application	6.8	3.1	16.0	3.6	9.7	9.6	2.6	4.1

**Table 8:** General results were obtained on Area 1 and Area 2 using each technique. - indicates no effectiveness, * low effectiveness, ** medium efficacy, and *** strong action.

	Area 1				Area 2			
Zones	VIS	UV	Spectrocolorimeter	ATP	VIS	UV	Spectrocolorimeter	ATP
2	*	*	-	*	-	*	*	*
3	*	-	-	-	-	-	-	-
4	**	**	***	*	**	**	***	*
5	-	**	-	-	-	-	-	-
6_1	**	*	*	*	***	*	**	*
6_2	-	-	**	-	-	*	-	-
7	-	-	-	-	*	-	-	-
8	*	-	-	-	**	*	*	-

## Data Availability

Not applicable.

## References

[1] Casaletto M.P., Privitera A., Testa M.L., La Parola V., Mazzaglia A., Zagami E.R. Hybrid β-cyclodextrin/silica systems for the preservation of biodeterioration of stone materials. Proceedings of the 9th European Conference on Ciclodextrines.

[2] Young M.E., Alakomi H.-L., Fortune I., Gorbushina A.A., Krumbein W.E., Maxwell I., McCullagh C., Robertson P., Saarela M., Valero J. (2008). Development of a biocidal treatment regime to inhibit biological growths on cultural heritage: BIODAM. Environ. Earth Sci..

[3] Crispim C., Gaylarde C. (2004). Cyanobacteria and biodeterioration of cultural heritage: A review. Microb. Ecol..

[4] Hrubec T.C., Seguin R.P., Xu L., Cortopassi G.A., Datta S., Hanlon A.L., Lozano A.J., McDonald V.A., Healy C.A., Anderson T.C. (2021). Altered toxicological endointsin humans from common quaternary ammonium compound disinfectant exposure. Toxicol. Rep..

[5] Zhang C., Cui F., Zeng G.-M., Jiang M., Yang Z.-Z., Yu Z.-G., Zhu M.-Y., Shen L.-Q. (2015). Quaternary ammonium compounds (QACs): A review on occurrence, fate and toxicity in the environment. Sci. Total Environ..

[6] Rotolo V., Barresi G., Di Carlo E., Giordano A., Lombardo G., Crimi E., Costa E., Bruno M., Palla F. (2016). Plant extract as green potential strategies to control the biodeterioration of cultural heritage. Int. J. Conserv. Sci..

[7] Fierascu I., Ion R.M., Radu M., Bunghez I.R., Avramescu S.M., Fierascu R.C. (2014). Comparative study of antifungal effects of natural extracts and essential oils. Rev. Roum. Chim..

[8] Cappitelli F., Cattò C., Villa F. (2020). The control of cultural heritage microbial deterioration. Microrganisms.

[9] Liu X., Koestler R.J., Warscheid T., Katayama Y., Gu J.-D. (2020). Microbial deterioration and sustainable conservation of stone monuments and buildings. Nat. Sustain..

[10] Ranalli G., Zanardini E. (2021). Biocleaning on cultural heritage: New frontiers of microbial biotechnologies. J. Appl. Microbiol..

[11] Kalemba D.A.A.K., Kunicka A. (2003). Antibacterial and antifungal properties of essential oils. Curr. Med. Chem..

[12] Fernandez F., Germinario S., Basile R., Montagno R., Kapetanaki K., Gobakis K., Kolokotsa D., Lagou A.M., Dania P., Enna M.T. (2020). Development of eco-friendly and self-cleaning lime-pozzolan plasters for bio-construction and cultural heritage. Buildings.

[13] Di Turo F., Medeghini L. (2021). How green possibilities can help in a future sustainable conservation of cultural heritage in Europe. Sustainability.

[14] Genova C., Fuentes E., Sanmartín P., Favero G., Prieto B. (2020). Phytochemical compounds as cleaning agents on granite colonized by phototrophic subaerial biofilms. Coatings.

[15] Warscheid T., Leisen H. (2011). Microbiological studies on stone deterioration and development of conservation measures at Angkor wat. Biocolon. Stone Control Prev. Methods.

[16] Siedenbiedel F., Tiller J.C. (2012). Antimicrobial polymers in solution and on surfaces: Overview and functional principles. Polymers.

[17] Salvadori O., Charola A.E. (2011). Methods to prevent biocolonization and recolonization: An overview of current research for architectural and archaeological heritage. Biocolon. Stone Control Prev. Methods.

[18] Rodrigues J.D., Anjos M.V., Charola A.E. (2011). Recolonization of marble sculptures in a garden environment. Biocolon. Stone Control Prev. Methods.

[19] Urzì C., De Leo F. (2007). Evaluation of the efficiency of water-repellent and biocide compounds against microbial colonization of mortars. Int. Biodeterior. Biodegrad..

[20] Galiano F., Mancuso R., Guzzo M.G., Lucente F., Gukelberger E., Losso M.A., Figoli A., Hoinkis J., Gabriele B. (2019). New polymeric films with antibacterial activity obtained by uv-induced copolymerization of acryloyloxyalkyltriethylammonium salts with 2-hydroxyethyl methacrylate. Int. J. Mol. Sci..

[21] Li H., Bao H., Bok K.X., Lee C., Li B., Zin M.T., Kang L. (2015). High durability and low toxicity antimicrobial coatings fabricated by quaternary ammonium silane copolymers. Biomater. Sci..

[22] Macchia A., Aureli H., Prestileo F., Ortenzi F., Sellathurai S., Docci A., Cerafogli E., Colasanti I.A., Ricca M., La Russa M.F. (2022). In-situ comparative study of eucaliptus, basil, cloves, thyme, pine and tea tree essential oils biocides efficacy. Methods Protoc..

[23] Si-Quat by AFFIX Labs, European Institute of Innovation and Technology, 8 September 2020. https://eit.europa.eu/our-activities/covid-19-response/solutions/si-quat-affix-labs.

[24] Monticello R.A., White W.C. (2010). White, inhibition of foundation colonization of biofilm by surface modification with organofunctional silanes. Applied Biomedical Microbiology: A Biofilms Approach.

[25] Randazzo L., Ricca M., Pellegrino D., La Russa D., Marrone A., Macchia A., Rivaroli L., Enei F., La Russa M.F. (2020). Anti-fouling additives for the consolidation of archaeological mortars in underwater environment: Efficacy tests performed on the apsidal fishpond of castrumnovum (Rome, Italy). Int. J. Conserv. Sci..

[26] BIOTERSUS Exentiae. 6 October 2019. https://www.exentiae.it/en/biotersus.

[27] Nakagawa S., Hillebrand G.G., Nunez G. (2020). Rosmarinus officinalis L. (Rosemary) extracts containing carnosic acid and carnosol are potent quorum sensing inhibitors of staphylococcus aureus virulence. Antibiotics.

[28] Endo E.H., Costa G.M., Makimori R.Y., Ueda-Nakamura T., Nakamura C.V., Dias Filho B.P. (2018). Anti-biofilm activity of rosmarinus officinalis, punicagranatum and tetradeniariparia against methicillin-resistant staphylococcus aureus (MRSA) and synergic interaction with penicillin. J. Herb. Med..

[29] Corbu V.M., Gheorghe I., Marinaș I.C., Geană E.I., Moza M.I., Csutak O., Chifiriuc M.C. (2021). Demonstration of allium sativum extract inhibitory effect on biodeteriogenic microbial strain growth, biofilm development, and enzymatic and organic acid production. Molecules.

[30] Koffi-Nevry R., Kouassi K.C., Nanga Z.Y., Koussémon M., Loukou G.Y. (2012). Antibacterial activity of two bell pepper extracts: Capsicum annuum L. and capsicum frutescens. Int. J. Food Prop..

[31] Essenzio IBIX BIOCARE. https://www.ibixbiocare.it/it/prodotti/essenzio.

[32] Rugnini L., Migliore G., Tasso F., Ellwood N.T.W., Sprocati A.R., Bruno L. (2020). Biocidal activity of phyto-derivative products used on phototrophic biofilms growing on stone surfaces of the domus aurea in Rome (Italy). Appl. Sci..

[33] Galiano F., Figoli A., Deowan S.A., Johnson D., Altinkaya S.A., Veltri L., De Luca G., Mancuso R., Hilal N., Gabriele B. (2015). A step forward to a more ecient wastewater treatment by membranesurface modification via polymerizable biocontinuous microemulsion. J. Membr. Sci..

[34] Mancuso R., Amuso R., Armentano B., Grasso G., Rago V., Cappello A.R., Galiano F., Figoli A., De Luca G., Hoinkis J. (2017). Synthesis and antibacterial activity of plymerizable acryloyloxytriethyl ammonium salts. ChemPlusChem.

[35] Marasco A., Nocerino S., Pinto G., Pollio A., Trojsi G., Natale A.D. (2016). Weathering of a Roman mosaic—A biological and quantitative study on in vitro colonization of calcareous tesserae by phototrophic microorganisms. PLoS ONE.

[36] DELTA®-LITE Dörken. https://www.doerken.com/it/Prodotti/Tetti/Membrane-traspiranti-e-altamente-traspiranti/delta-lite.php.

[37] Macchia A., Biribicchi C., Carnazza P., Montorsi S., Sangiorgi N., Demasi G., Prestileo F., Cerafogli E., Colasanti I.A., Aureli H. (2022). Multi-analytical investigation of the oil painting “*Il Venditore di Cerini*” by antonio mancini and definition of the best green cleaning treatment. Sustainability.

[38] Macchia A., Biribicchi C., Rivaroli L., Aureli H., Cerafogli E., Colasanti I.A., Carnazza P., Demasi G., La Russa M.F. (2022). Combined use of non-invasive and micro-invasive analytical investigations to understand the state of conservation and the causes of degradation of I tesori del mare (1901) by Plinio Nomellini. Methods Protoc..

[39] Sanmartin P., Vázquez-Nion D., Silva B., Prieto B. (2012). Spectrophotometric color measurement for early detection and monitoring of greening on granite buildings. Biofouling.

